# Epidural analgesia and its implications in the maternal health in a low parity comunity

**DOI:** 10.1186/s12884-019-2191-0

**Published:** 2019-01-30

**Authors:** Ivan Penuela, Pilar Isasi-Nebreda, Hedylamar Almeida, Mario López, Esther Gomez-Sanchez, Eduardo Tamayo

**Affiliations:** 1 Unit of Anesthesiology, Resuscitation and Pain Treatment, University Hospital Joan XXIII, Carrer Dr. Mallafré Guasch, 4, Tarragona, Spain; 2 Unit of Anesthesiology, Resuscitation and Pain Treatment, University Hospital of Burgos, Burgos, Spain; 3 Unit of Anesthesiology, Resuscitation and Pain Treatment, Clinic University Hospital, Valladolid, Spain

**Keywords:** Epidural analgesia, Perineal tears, Caesarean section, Delivery, Labor, Maternal outcomes

## Abstract

**Background:**

In regard to obstetrical analgesia management there are different results related to the use of epidural analgesia versus mechanical adverse outcomes at delivery.

**Methods:**

Cohort study of 23,183 consecutive, term, singleton vaginal deliveries, including spontaneous and induced labours, at a single institution from January 2004 to June 2016 to determine the association between epidural analgesia and different mechanical complications affecting maternal health such as severe perineal tears (SPT), abnormal foetal head position at delivery, instrumental delivery and caesarean section (CS).

Multivariate logistic regression models were constructed to evaluate the risk factors of these mechanical complications with respect to possible cofounders.

**Results:**

Epidural analgesia was used in 15,821 (68.24%) women. The logistic regression model showed a non-significant association between the use of epidural analgesia and SPT (odds ratio [OR], 078; 95% confidence interval [CI], 0.48–1.26; *p* = 0.310). Instrumental delivery and CSs were more frequently performed in cases than controls (*p* = < 0.001), with OR of 1.19 (95% CI: 1.10–1.29) for CS and with OR of 3.27 (95% CI: 2.93–4.61) for instrumental delivery. The abnormal foetal position head at delivery were significantly lower in the neonates delivered without epidural analgesia compared with those in which epidural analgesia was used (*p* < 0.001) with OR of 1.43 (95% CI:1.27–1.72).

**Conclusions:**

Epidural analgesia is not associated with an increase of SPT, but it was an independent risk factor for instrumental delivery, CS and abnormal foetal head position at delivery.

## Background

Epidural analgesia is one of the best procedures for pain management strategy during labour. Despite its effectiveness, the use of epidural anaesthesia presents risks.

Maternal changes induced by epidural analgesia during labour may affect the mother and baby. Hypotension, fever, prolonged labour and delivery, an increased need for oxytocin, and instrumental delivery have been associated with epidural analgesia. Its use has also been linked with dystocia [1–3]. Patients using epidurals are more likely to require oxytocin for labour augmentation, have prolonged second stages of labour and show persistent occipitoposterior foetal malposition [4–9].

Instrumental delivery is associated with severe perineal tears (SPT) in a good percentage of vaginal deliveries, resulting in short- and long-term perineal pain, dyspareunia, urinary incontinence, voiding and defecatory dysfunction, as well as urinary and bowel incontinence (fecal or gas incontinence or both). SPT, defined as third- and fourth-degree perineal lacerations, appear to have an immediate impact on pelvic floor muscle function.

Different studies have shown the association between epidural analgesia and an increased number of instrumental deliveries [10–14]. Other authors have also found a relationship between epidural analgesia and a major incidence of caesarean delivery [15–17].

One study has found a correlation among epidural analgesia, instrumental delivery, and SPT [13], but in this study midline episiotomy was performed, whereas in our hospital mediolateral episiotomies are far more commonly performed. This can explain as well why in our study there are fewer mechanical complications. Another study [12] initially found an association between epidural analgesia and SPT, but when instrumental delivery was included in their model, the use of epidural analgesia has not shown a statistically significant difference.

The aim of this study was to determine the association between epidural analgesia and different mechanical complications affecting maternal health such as SPT**,** instrumental delivery, caesarean section (CS), and abnormal foetal head position at delivery.

## Methods

We studied all patients who underwent operative vaginal delivery from January 2004 to December 2016 in the University Hospital of Burgos, Spain. The University Hospital of Burgos is a tertiary hospital and its Obstetrics Department is the largest its province, with over 2000 deliveries performed annually.

In our Hospital, the rate of epidural analgesia during labor is greater than 60%, a percentage that is very similar to Hospitals in the USA and higher than other European Hospitals, since we have a full time anaesthesiologist available for the Obstetrics department and we have not received complaints from this Department as far as the length of labor is concerned, when epidural analgesia is provided.

Epidural analgesia is administered to the patient upon Obstetrics department request, when the patient has nothing that contra-indicates its use, normally when cervix is approximately 4 to 5 cm dilated (late epidural analgesia). We deliver 10 ml bolus of Ropivacaine 0.2%™ plus Fentanyl™ 50 mcg, and then a continuous Ropivacaine 0.2% plus Fentanyl™ 1 mcg/ml infusion (6-10 ml/h) or an initial bolus of 10 ml Levobupivacaine 0.125%™ plus 50 mcg Fentanyl™, followed by continuous infusion of Levobupivacaine 0.0625%™ plus 1mcg/dl Fentanyl™ (6-10 ml/l); both options can be followed by intermittent perfusion of 5 ml with 20 min lockout time.

Women were included in this study if they were admitted with a singleton pregnancy and delivered vaginally. Exclusion criteria included multiple gestations, elective caesarean section, preeclampsia, gestational diabetes or preterm delivery (defined as gestational age of less than 37 weeks), as well as home deliveries or births not occurring on a labour and delivery unit (out of hospital settings).

The information necessary for the present work was collected from the medical records and consigned in a database designed for that purpose. The name of the patients and other data that could individually identify each pregnant woman were changed to numerical codes.

Since the present study is consisted of an anonymous retrospective analysis and due to the non-identification of patients, there was no need for individual informed consent from patients.

The study protocol was submitted to the ethic committee for clinical research of the University Hospital of Burgos and was exempted on the basis of an anonymous analysis.

### Statistical analysis

Data comparison was made by using Microsoft Excel and Statistical Package for Social Sciences (SPSS) version 23.0 (SPSS Inc., Chicago, IL) as appropriate. Group differences in categorical outcome variables were assessed using Chi-squared or Fisher exact tests. Yates’ correction was applied to tables with one or more cells with expected frequency less than five. Continuous variables were examined using the 2-sample t-tests using Satterwaite correction in cases with different variables. The association between epidural analgesia and some mechanical complications (severe perineal tears, instrumental delivery, CS, and abnormal foetal head position) was studied using multivariable logistic regressions controllled for the study covariates. We report odds ratios (OR) and 95% confidence intervals (95% CI). Two-sided *p* values less than 0.05 were considered statistically significant.

## Results

We included 23,183 singleton vaginal deliveries in our study. The demographic characteristics of the study population by analgesia during labour are shown in Table 1.

**Table 1:** Maternal characteristics by use of epidural analgesia

Maternal characteristic	Epidural analgesia Yes *n* (%) 15,821 (68.24)	Epidural analgesia No *n* (%) 7362 (31.76)	*p* value
Age	31.72 ± 4.99	32.18 ± 5.15	< 0.001
Gestational age at birth	39.42 ± 1.10	39.21 ± 1.11	< 0.001
Parity 1	9825 (62.1)	2417 (32.8)	< 0.001
Parity 2	5025 (31.7)	3664 (49.7)	< 0.001
Parity 3 and more	979 (6.1)	1281 (17.4)	< 0.001
Induction	224 (1.4)	166 (2.2)	< 0.001
Spontaneous	15,597 (98.5)	7196 (97.7)	< 0.001
New-born Weight	3280 ± 420	3004 ± 670	< 0.001
Severe perineal tears	63 (0.40)	28 (0.38)	0.310
Abnormal foetal head position at delivery	522 (3.30)	150 (2.04)	< 0.001
Instrumental delivery	3582 (22.64)	370 (5.03)	< 0.001
Episiotomy	9799 (52.9)	3356 (45.5)	< 0.001
Caesarean delivery	2544 (16.08)	1017 (13.81)	< 0.001
New-born male	8337 (52.69)	3755 (51.00)	0.01

The data are expressed as median ± SD (Standard Deviation), absolute rate and percentage. Statistical significance was defined as *p*-value < 0.05

On the one hand, 1387 women (18.83%) in the group of non-epidural analgesia didn’t suffer any sort of mechanical complications whatsoever. On the other hand, in the group of epidural analgesia 6064 (39.68%) women did not suffer any mechanical complications.

Epidural analgesia was more frequently administered to lower parity patients (62.1 versus 32.8%). Basically, SPT was an observed complication in 0.4% (*n* = 91) of the cases; as for the multivariate analyses, parity was inversely associated with sever tears (OR of 0.029 for parity of 1, versus parity of 2 or more). There was a strong association between SPT and instrumental delivery with OR of 2.97. Furthermore, birth weight was positively associated with SPT with OR of 3.53.

In our study population, 68.24% (*n* = 15,821) of the patients was administered epidural analgesia, SPT complicated 0.4% (*n* = 63) of births in which epidural analgesia was administered, compared with 0.38% (*n* = 28) of births where no epidural was administered, yielding an OR of 1.05 (95% CI: 0.67–1.64). Our first multivariable model included epidural analgesia, parity, birth weight, instrumental delivery, episiotomy and sex of the new born, In this model, parity (OR: 3.71, 95% IC: 1.14–12.00), instrumental delivery (OR: 2.97, 95% IC: 1.79–4.92) and birth weight (OR: 3.53, 95% IC: 1.81–6.90) were found to be the most significant risk factor for SPT, Epidural analgesia was no found to be a risk factor for SPT (OR: 1.05, 95% CI: 0.69–1.29) (Table 2).

**Table 2:** Univariate and multivariate logistical models of predictors of severe perineal tears (SPT)

Maternal characteristic		SPT	Univariate analysis OR (95% IC)	*p* value	SPT	Multivariate analysis OR (95% IC)	*p* value
Epidural analgesia	Yes	63/15758 (0.40)	1.05 (0.67–1.64)	0.826	63/15758 (0.40)	0.780 (0.48–1.26)	0.310
	No	28/7360 (0.38)	Ref.		28/7360 (0.38)	Ref.	
Age	Yes	32.85 ± 4.05		0.895	32.85 ± 4.05	0.824 (0.76–1.32)	0.721
	No	31.86 ± 5.05	Ref.		31.86 ± 5.05		
Parity	Parity 1	58/12242 (0.47)	1.37 (0.88–2.13)	0.152	58/12242 (0.47)	3.71 (1.14–12.00)	0.029
	Parity 2	30/8681 (0.35)	2.95 (0.70–12.38)	0.131	30/8681 (0.35)	Ref.	
	3 or more	3/2260 (0.13)	4.05 (0.99–16.63)	0.051	3/2260 (0.13)	Ref.	
Instrumental delivery	Yes	35/3938 (0.89)	2.50 (1.63–3.82)	< 0.001	35/3938 (0.89)	2.97 (1.79–4.92)	< 0.001
	No	56/19245 (0.29)	Ref.		56/19245 (0.29)	Ref.	
Episiotomy	Yes	62/13155 (0.47)	1.63 (1.04–2.53)	0.023	62/13155 (0.47)	0.67 (0.40–1.13)	0.139
	No	29/10028 (0.29)	Ref.		29/10028 (0.29)	Ref.	
New-born male	Yes	39/12092 (0.32)	0.68 (0.45–1.04)	0.07	39/12092 (0.32)	0.65 (0.42–1.01)	0.056
	No	52/11091 (0.47)	Ref.		52/11091 (0.47)	Ref.	
Weight > 4000 g,	Yes	10/1070 (0.93)	2.57 (1.32–4.96)	0.005	10/1070 (0.93)	3.53 (1.81–6.90)	< 0.001
	No	81/22113 (0.37)	Ref.		81/22113 (0.37)	Ref.	

Subsequent analyses which included parity, weight of birth, episiotomy, sex of the new-born and episiotomy; instrumental delivery, parity and weight of birth were each found to be independently and similarly associated with SPT with OR of 2.97 (95% CI: 1.79–4.92), 3.71 (95% CI: 1.14–12.00), and 3.53 (95% CI: 1.81–6.90), respectively, Epidural analgesia was not a risk factor for SPT (OR: 0.78, 95% CI: 0.48–1.26),

The differences in neonatal outcomes between cases and controls, stratified by the mode of delivery, are shown in Tables 3 and 4, Instrumental delivery and CS were more frequently performed in cases than controls (*p* = < 0.001), with OR of 1.19 (95% CI: 1.10–1.29) for CS and with OR of 3.27 (95% CI: 2.93–4.61) for instrumental delivery,

**Table 3:** Univariate and Multivariate logistical models of predictors of caesarean delivery

Maternal characteristic		Caesarean delivery	Univariate analysis OR (95% IC)	*p* value	Caesarean delivery	Multivariate analysis OR (95% IC)	*p* value
Epidural analgesia	Yes	2544/15821 (16.08)	1.19 (1.10–1.29)	< 0.001	2544/15821 (16.08)	1.19 (1.10–1.29)	< 0.001
	No	1017/7362 (13.81)	Ref.		1017/7362 (13.81)	Ref.	
Age	Yes	32.14 ± 4.08		0.723	32.14 ± 4.08		0.823
	No	31.75 ± 3.96	Ref.		31.75 ± 3.96	Ref.	
Parity	Parity 1	2478/12242 (20.24)	2.13 (1.99–2.35)	< 0.001	2478/12242 (20.24)	3.71 (1.14–12.00)	< 0.001
	Parity 2	909/8681 (10.47)	2.95 (0.70–12.38)	0.131	909/8681 (10.47)	Ref.	
	3 or more	174/2260 (7.69)	4.05 (0.99–16.63)	0.051	174/2260 (7.69)	Ref.	
Episiotomy	Yes	256/13155 (1.94)	0.04 (0.03–0.04)	< 0.001	256/13,155 (1.94)	0.04 (0.03–0.04)	< 0.001
	No	3305/10028 (32.95)	Ref.		3,05/10028 (32.95)	Ref.	
New-born male	Yes	2005/12092 (16.58)	1.21 (1.13–1.30)	< 0.001	2005/12092 (16.58)	1.21 (1.13–1.30)	0.056
	No	1556/11091 (14.02)	Ref.		1556/11091 (14.02)	Ref.	
Weight > 4000 g,	Yes	299/1070 (27.94)	2.24 (1.95–2.57)	< 0.001	299/1070 (27.94)	2.24 (1.95–2.57)	< 0.001
	No	3262/22113 (14.75)	Ref.		3262/22113 (14.75)	Ref.

**Table 4:** Univariate and Multivariate logistical models of predictors of instrumental delivery

Maternal characteristic		Instrumental delivery	Univariate analysis OR (95% IC)	*p* value	Instrumental delivery	Multivariate analysis OR (95% IC)	*p* value
Epidural analgesia	Yes	3582/15821 (22.64)	4.50 (4.03–5.03)	< 0.001	3582/15821 (22.64)	3.27 (2.93–4.61)	< 0.001
	No	370/7362 (5.03)	Ref.		370/7362 (5.03)	Ref.	
Age	Yes	31.72 ± 4.14		0.798	31.72 ± 4.14		0.714
	No	31.13 ± 3.86	Ref.		31.13 ± 3.86	Ref.	
Parity	Parity 1	2988/12242 (24.40)	1.37 (0.88–2.13)	0.152	2988/12242 (24.40)	3.71 (1.14–12.00)	< 0001
	Parity 2	950/8681 (10.94)	2.95 (0.70–12.38)	0.131	950/8681 (10.94)	Ref.	
	3 or more	14/2260 (0.52)	4.05 (0.99–16.63)	0.051	14/2260 (0.52)	Ref.	
Episiotomy	Yes	3858/13155 (29.32)	51.59 (41.27–64.49)	< 0.001	3858/13155 (29.32)	51.59 (41.27–64.49)	0.155
	No	80/10026	Ref.		80/10026	Ref.	
New-born male	Yes	2163/12092 (17.88)	1.22 (1.13–1.30)	< 0.001	2163/12092 (17.88)	1.22 (1.13–1.30)	0.012
	No	1775/11091 (16.00)	Ref.		1775/11091 (16.00)	Ref.	
Weight > 4000 g,	Yes	157/1070 (14.67)	0.83 (0.70–0.99)	0.039	157/1070 (14.67)	0.83 (0.70–0.99)	< 0.001
	No	3781/22113 (17.09)	Ref.		3781/22113 (17.09)	Ref.	

The abnormal foetal position head at delivery were significantly lower in the neonates delivered without epidural analgesia compared with those in infants by epidural analgesia, (*p* < 0.001) with OR of 1.43 (95% CI:1.27–1.72), these results are shown in Table 5,

**Table 5:** Univariate and Multivariate logistical models of predictors of abnormal foetal head position at delivery

Maternal characteristic		Abnormal foetal position head al delivery	Univariate analysis OR (95% IC)	*p* value	Abnormal foetal position 'head al delivery	Multivariate analysis OR (95% IC)	*p* value
Epidural analgesia	Yes	522/15821 (3.30)	1.64 (1.36–1.97)	< 0.001	522/15821 (3.30)	1.43 (1.27–1.72)	< 0.001
	No	150/7362 (2.04)	Ref.		150/7362 (2.04)	Ref.	
Age	Yes	32.78 ± 4.08		0.863	32.85 ± 4.05	0.824 (0.76–1.32)	0.825
	No	31.91 ± 5.10	Ref.		31.86 ± 5.05		
Parity	Parity 1	58/12242 (0.47)	1.37 (0.88–2.13)	0.152	58/12242 (0.47)	3.71 (1.14–12.00)	0.125
	Parity 2	30/8681 (0.35)	2.95 (0.70–12.38)	0.131	30/8681 (0.35)	Ref.	
	3 or more	3/2260 (0.13)	4.05 (0.99–16.63)	0.051	3/2260 (0.13)	Ref.	
Instrumental delivery	Yes	11/441 (2.49)	0.12 (0.06–0.22)	< 0.001	11/441 (2.49)	0.12 (0.06–0.22)	< 0.001
	No	3927/22742 (17.26)	Ref.		3927/22742 (17.26)	Ref.	
Episiotomy	Yes	92/441 (20.86)	0.19 (0.15–0.24)	< 0.001	92/441 (20.86)	0.19 (0.15–0.24)	0.148
	No	13,063/22742 (57.43)	Ref.		13,063/22472 (57.43)	Ref.	
Newborn masculine	Yes	224/12092 (1.85)	0.94 (0.74–1.14)	0.562	224/12092 (1.85)	0.94 (0.74–1.14)	0.780
	No	217/11091 (1.95)	Ref.		217/11091 (1.95)	Ref.	
Weight > 4000 g,	Yes	10/1070 (0.93)	0.47 (0.25–0.89)	0.020	10/1070 (0.93)	0.47 (0.25–0.89)	< 0.051
	No	431/22113 (1.94)	Ref.		431/22113 (1.94)	Ref.	

## Discussion

The most relevant outcomes in this present study are: epidural analgesia is not an independent risk factor for SPT, but it is for instrumental delivery, CS and abnormal foetal head position at delivery.

Lowemberg et al. [11], who did a retrospective cohort study during a period between 2006 and 2011, in 61,308 women during labour in which 31,631 received epidural analgesia; severe perineal tear was found in 0.3% at delivery. Births with epidural analgesia had a higher rate of primiparity, augmentation of labour, instrumental delivery and episiotomy. They concluded that epidural analgesia was not related with severe perineal tears once confounding variables were controlled. In our study, epidural analgesia was not associated with an increased risk for SPT, just like the outcomes and conclusions previously reported in previous, similar studies [7, 11, 14].

Simhan et al. [13] studied risk factors for rectal lesion after the delivery to determine the impact according with the experience of who assisted the labour. 17,722 women were included in that study. Rectal damage was found in 8.9% (*n* = 1.572). They also found a relation between episiotomy tear and SPT, with midline episiotomy whilst in our hospital it is performed almost 100% middle laterally.

To define the possible collateral effects in short and long term between epidural analgesia and non-epidural analgesia to relief labour pain, C.J Howell et al. [10] designed a random controlled study, in 369 women at their first birth. Among other results they found that the incidence of instrumental delivery was higher in the group that received epidural analgesia (30% vs 19%, OR: 1.77, 95 CI: 1.09–2.86). Instead of the great proportion of women in each group that did not receive analgesia, a significant difference in terms of instrumental delivery remained.

Also to clarify if the adverse results in short term were associated with epidural analgesia itself or with instrumental delivery, Hasegawa et al. [18] performed a retrospective case control study to evaluate the relationship between epidural analgesia, labour length y perinatal results in 350 women under epidural analgesia, compared to 1400 women without epidural analgesia. Instrumental delivery was more frequent in women with epidural analgesia (6.5 vs 2.9%). Therefore they concluded that epidural analgesia is associated with low progression of labour and more instrumental delivery. Our study showed that epidural analgesia was associated with major probability of instrumental delivery, in some studies this finding was related to epidural analgesia and others studies did not establish a direct correlation between epidural analgesia and instrumental delivery [7, 8, 12, 14, 19–24].

To evaluate if epidural analgesia is associated with a higher rate of abnormal foetal position, Lieberman et al. [6] conducted a retrospective cohort study in 1562 women to evaluate the changes of the foetal position during labour by using ultrasonography. Women with epidural analgesia did not have more foetuses in occipito posterior in the recruitment (23.4% epidural vs. 28.3% non-epidural), but had more foetuses in occipito posterior at the birth (12. 9% epidural vs. 3.3% non-epidural, *p* = 0.002); this association remained in the multivariate model (OR: 4.0 95% CI: 1.4–11.1). Our study agrees with the results of this above mentioned study, concluding that the abnormal foetal position head at delivery were significantly lower in the neonates delivered without epidural analgesia compared with those in infants born under the effects of epidural analgesia [6]**.** Therefore epidural analgesia is associated to a higher observation of abnormal presentations of the new-born, although we also observed that this is not the only risk factor, because primiparity is associated to abnormal presentations as well.

To compare the effects of the epidural analgesia with intravenous analgesia in labour, Ramin et al. [15] randomized women with no complicated labour to offer them epidural analgesia with bupivacaine or intravenous analgesia with fentanilo or meperidine. 437 women accepted meperidine and were compared with 432 women who accepted epidural analgesia.

Epidural analgesia produced better pain relief when compared to intravenous meperidine, nevertheless it has also increased the risk of CS from 2 to 4.

On the other hand, Sharma et al. [16] developed a metaanalisis of 2703 women who were randomized to epidural analgesia or intravenous opioids pain management in labour, in five clinical trials in their hospitals. There were no difference in the rate of CS between the two groups (epidural analgesia 10.5% vs. intravenous analgesia 10.3%), OR: 1.04 95% CI: 0.81–1.84; *p* = 0.920). They concluded that epidural analgesia during labour does not increase the number of CS. In our study CSs were more frequently performed in cases than controls similar to others studies [15, 25–30], but different to others studies that did not find association between analgesia epidural and intravenous analgesia for CS [16, 31] or others when association between epidural analgesia and CS was not found [17].

The difference between the study of Sharma and our results could be because our study compares epidural analgesia with no analgesia.

In our study CSs were more frequently performed in cases than controls similar to others studies [25–30], but different to other studies in which no association between analgesia epidural and intravenous analgesia for CS was found [32] or others in which the association between epidural analgesia and CS was not observed [17].

Our study was able to demonstrate that primiparity and new-born weight over 4000 g are significant risk factors for SPT, similarly to other studies [11, 20]. Both variables are considered biological risk factors for SPT development, as well as for frequent indication factors for epidural analgesia use, which could possibly mislead to a wrong association between epidural analgesia and SPT. Furthermore, our study shows that primiparity is a risk factor for CS, instrumental delivery and abnormal foetal head position at delivery and similarly the new-born weight over 4000 g ended up also being considered as a risk factor for CS, instrumental delivery but not for abnormal foetal head position at delivery. There is a need for further studies in order to establish the specific weight of these risk factors into leading to the development of mechanical adverse outcomes at delivery.

The present study supports with more evidence the correlation between epidural analgesia and mechanical complications during labour in women with low parity, and clarifies the weight of the different risk factor of these mechanical complications. Thus it is indeed a good tool for physicians when they have to decide when and how to use this important resource in their daily clinical routine.

As we have mentioned previously, the present work was carried out entirely in the University Hospital of Burgos, for which multicentric studies would be necessary to support or refute our findings. On the other hand, the population size that we have analyzed gives strength to the results found about the involvement of epidural analgesia with respect to the adverse mechanical outcomes at delivery.

## Conclusion

In conclusion, we have found that epidural analgesia is a safe method for pain relief during labour, and it is not associated with an increase of SPT, but it was an independent risk factor for instrumental delivery, CS and abnormal foetal head position at delivery.

New-born weight over 4000 g and primiparity are independent risk factors for SPT, instrument delivery and CS procedure. As for this last one, it was also considered a risk factor for abnormal foetal head position at delivery.

Notwithstanding, we should not avoid the administration of epidural analgesia for fear of increased risk of SPT and its complications. It is necessary to evaluate the specific weight of other risk factors in SPT development and other mechanical adverse outcomes at delivery.
